# Correlation between municipal human development index and stroke mortality: a study of Brazilian capitals

**DOI:** 10.1186/s13104-018-3626-9

**Published:** 2018-08-01

**Authors:** Diego Monteiro de Melo Lucena, Francisco Winter dos Santos Figueiredo, Luiz Vinicius de Alcantara Sousa, Laércio da Silva Paiva, Tábata Cristina do Carmo Almeida, Sidnei José Galego, João Antônio Correa, Erika da Silva Maciel, Fernando Adami

**Affiliations:** 1 Laboratório de Epidemiologia e Análise de Dados, Faculdade de Medicina do ABC, Santo André, São Paulo Brazil; 2 Disciplina de Angiologia e Cirurgia Vascular, Faculdade de Medicina do ABC, Santo André, Brazil; 3 Universidade Federal do Tocantins, Palmas, Brazil

**Keywords:** Stroke, Epidemiology, Socioeconomic status

## Abstract

**Objective:**

To analyze the correlation between municipal human development indices (MHDIs) and stroke mortality in residents of Brazilian state capitals in 2010. A secondary data analysis was conducted in 2015 using data for the MHDI and the following dimensions: income, longevity and education which were obtained from the United Nations Development Program. Additionally, we analyzed age-standardized stroke mortality data from the Department of System Information Unified Health of Brazil.

**Results:**

We observed a correlation between stroke mortality and MHDIs overall (Pearson r = − 0.563; p = 0.002) and within the following dimensions: income (Spearman’s ρ = − 0.479; p = 0.011), longevity (Pearson r = − 0.510; p = 0.006) and education (Pearson r = − 0.592; p = 0.001). We identified moderate but significant negative correlations between MHDI overall and in its individual dimensions (income, longevity, and age) and stroke mortality in Brazilian capitals. Stroke is the second leading cause of death in industrialized countries and the leading cause of death in Brazil. Therefore, the discovery of factors that may influence the epidemiology of stroke is important for the construction of adequate policies considering to the socioeconomic status in these places and with an emphasis in lower socioeconomic status places.

## Introduction

Each year, strokes are suffered by approximately 15 million people worldwide. Of these persons, approximately 5 million die [1]; it is estimated that by 2030, stroke may represent the sixth leading cause of disability-adjusted life-years lost in the world [2, 3]. Although the number of strokes is alarming, a gradual decrease in the occurrence of stroke has been observed in developed countries. This gradual decrease is likely due to better systemic arterial hypertension (SAH) control and a decrease in the levels of smoking in these populations [1].

Most fatal stroke cases occur among men aged from 45 to 59 years who live in countries with lower levels of socioeconomic development, such as Caribbean countries and former socialist countries in Europe [4]. In Brazil, a country that is considered developing [5], the prevalence of stroke has decreased. However, the disease rate has not decreased as fast as it has in developed nations [6, 7].

Studies of the association between socioeconomic status and stroke risk in adults have shown conflicting conclusions, demonstrating both positive and negative correlations [8, 9]. Recent studies [10, 11] regarding the effect of different socioeconomic statuses throughout the life span have shown that children of lower socioeconomic status may be at higher risk of stroke in adulthood. In parallel, higher stroke prevalence rates have been observed in African countries than in developed nations [12].

Human development indexes (HDIs) represent one mechanism by which to analyze socioeconomic status and are also the most frequently used indicator of the socioeconomic level of a nation [13]. These indexes are in-depth measures of not only the economic profile but also quality of life, longevity, health services and education in a nation [13]. However, few studies have analyzed the role of HDI in the epidemiology of stroke both in Brazil and worldwide [4].

Although Brazil has a large population and vast geographic plurality, few studies have been conducted to generate understanding regarding the effect of differences in income distribution, education and longevity on health indicators [14]. One 2013 study [4] also identified a correlation between stroke mortality and HDIs in the global context. Analyses using the HDI dimensions may serve as important instruments in understanding the associations between mortality, income, longevity and education.

Thus, the objective of this study was to analyze the correlations between MHDIs and its dimensions for income, education and longevity with the stroke mortality in Brazilian state capitals.

## Main text

### Materials and methods

A secondary data analysis was conducted using municipal human development index (MHDI) and stroke mortality data for Brazilian state capitals.

HDI data from Brazilian state capitals in 2010 was collected from the United Nations Development Program (UNDP—http://www.pnud.org.br). The UNDP website, which is maintained by the United Nations, includes a virtual atlas that may be used to analyze and quantify the rate of development in municipalities.

The HDI is calculated based on indicators of living on a healthy and long life (longevity); access to knowledge, as measured by various factors (education); and standard of living, as indicated by Gross National Income [15]. We decided to analyze MHDI data from only 2010 due the implementation of a new calculation method and non-availability data after this year.

Stroke mortality data were collected from the Mortality Information System (*Sistema de Informação de mortalidade*—*SIM*) of the Department of the Brazilian Unified Health System Information (DATASUS—http://www.datasus.gov.br), which is a demographic, social and health database maintained by the Brazilian Ministry of Health.

Stroke was defined according to International Diseases Classification codes (10th edition; ICD-10) (OMS 1997) and included subarachnoid hemorrhage (I60), intracranial hemorrhage (I61), cerebral infarction (I63) and stroke not specified as ischemic and/or hemorrhagic (I64).

The gross stroke mortality rate was calculated by dividing the number of deaths due to stroke in Brazilian capitals by the total population living in each capital and multiplying the result by 100,000 inhabitants, and this measure was then standardized by the age of the population using the direct standardization method of the World Health Organization [16].

Data were independently collected using data collection forms by two different researchers. Subsequently, the data were validated, and the disagreements were independently resolved by a third researcher.

Descriptive statistics were performed, and absolute and relative frequencies were calculated. Spearman’s (ρ) correlation tests were performed on the MHDI income variable, as it did not demonstrate a normal distribution (Shapiro–Wilk test, p < 0.05), and Pearson’s correlation (r) was used for the MHDI dimensions, education and longevity, as the evaluated data demonstrated normality, as indicated by the Shapiro–Wilk test (p > 0.05). The confidence level was 95%. Stata 11.0 was used for the statistical analyses.

### Results

Table 1 describes stroke age-standardized mortality (per 100,000 inhabitants), MHDI and its dimensions (income, education and longevity). We observed higher rates of age-standardized stroke mortality in southeastern region capitals. We also observed higher HDIs and income, longevity and education in southern region capitals.

**Table 1:** Description of stroke age-standardized mortality (per 100,000 inhabitants), MHDI and its dimensions by capitals of each region

Capital per region	Stroke age-standardized mortality (per 100,000 inhabitants)	MHDI			
		MHDI	Income	Education	Longevity
North					
Porto Velho	48.60	0.736	0.764	0.638	0.819
Rio Branco	47.78	0.727	0.729	0.661	0.798
Manaus	33.67	0.737	0.738	0.658	0.826
Boa Vista	36.33	0.752	0.737	0.708	0.816
Belem	55.39	0.746	0.751	0.673	0.822
Macapá	46.34	0.733	0.723	0.633	0.820
OPalmas	39.57	0.788	0.789	0.749	0.827
Northeast					
São Luis	38.55	0.768	0.741	0.752	0.813
Teresina	38.50	0.751	0.731	0.707	0.820
Fortaleza	29.60	0.754	0.749	0.695	0.824
Natal	25.99	0.763	0.768	0.694	0.835
João Pessoa	38.47	0.763	0.770	0.693	0.832
Recife	27.09	0.772	0.798	0.698	0.825
Maceió	43.44	0.721	0.739	0.635	0.799
Aracaju	27.28	0.770	0.784	0.708	0.823
Salvador	35.20	0.759	0.772	0.679	0.835
Southeast					
Belo Hoiizonte	30.69	0.810	0.841	0.737	0.856
Vitória	32.69	0.845	0.876	0.805	0.855
Rio de Janeiro	34.79	0.799	0.840	0.719	0.845
São Paulo	37.98	0.805	0.843	0.725	0.855
South					
Curitiba	26.88	0.823	0.850	0.768	0.855
Florianópolis	20.45	0.847	0.870	0.800	0.873
Porto Alegre	43.86	0.805	0.867	0.702	0.857
Center West					
Campo Grande	35.63	0.784	0.790	0.724	0.844
Cuiabá	38.44	0.785	0.800	0.726	0.834
Goiânia	30.99	0.799	0.824	0.739	0.838
Brasilia	32.07	0.824	0.863	0.742	0.873

In addition, there were some capitals that stood out as having higher stroke mortality rates than other capitals in the same region. In the southern region, Porto Alegre, which had an MHDI of 0.805 (55.51 deaths per 100,000 inhabitants), had approximately twice the age-standardized mortality rate as other capitals in the same region. The other capitals in the same region, Curitiba and Florianópolis, had MHDIs of 0.823 and 0.847, respectively.

Furthermore, in the northern region, Belém stood out due to its age-standardized mortality rate of 42.47 per 100,000 inhabitants. Some other capitals in the northern region, Porto Velho, Rio Branco and Macapá, had substantial age-standardized mortality rates of 48.6, 47.78, and 46.34, respectively.

In the northeastern region, Maceió stood out as having a higher age-standardized mortality rate (43.44) and lower HDI overall (0.721) and within the income (0.739), education (0.635) and longevity (0.799) dimensions. On the other hand, Recife, which is also located in the northeastern region, has a low rate of age-standardized mortality (27.09) and a high HDI (0.772) overall and in the dimensions for income (0.798) and longevity (0.825).

Capitals in the southeastern region did not demonstrate large disparities in the association between mortality and HDI. However, Vitória had higher socioeconomic development and a lower age-standardized stroke mortality rate (32.69) when compared with São Paulo (37.98) and Rio de Janeiro (34.79), some of the most populated cities in Brazil.

In the central western region, Goiânia and Brasília had lower mortality rates (30.99 and 32.07, respectively) and HDIs (0.799 and 0.824, respectively). Cuiabá had a higher mortality rate (38.44) and, curiously, the lowest longevity MHDI (0.834).

**Fig. 1:**
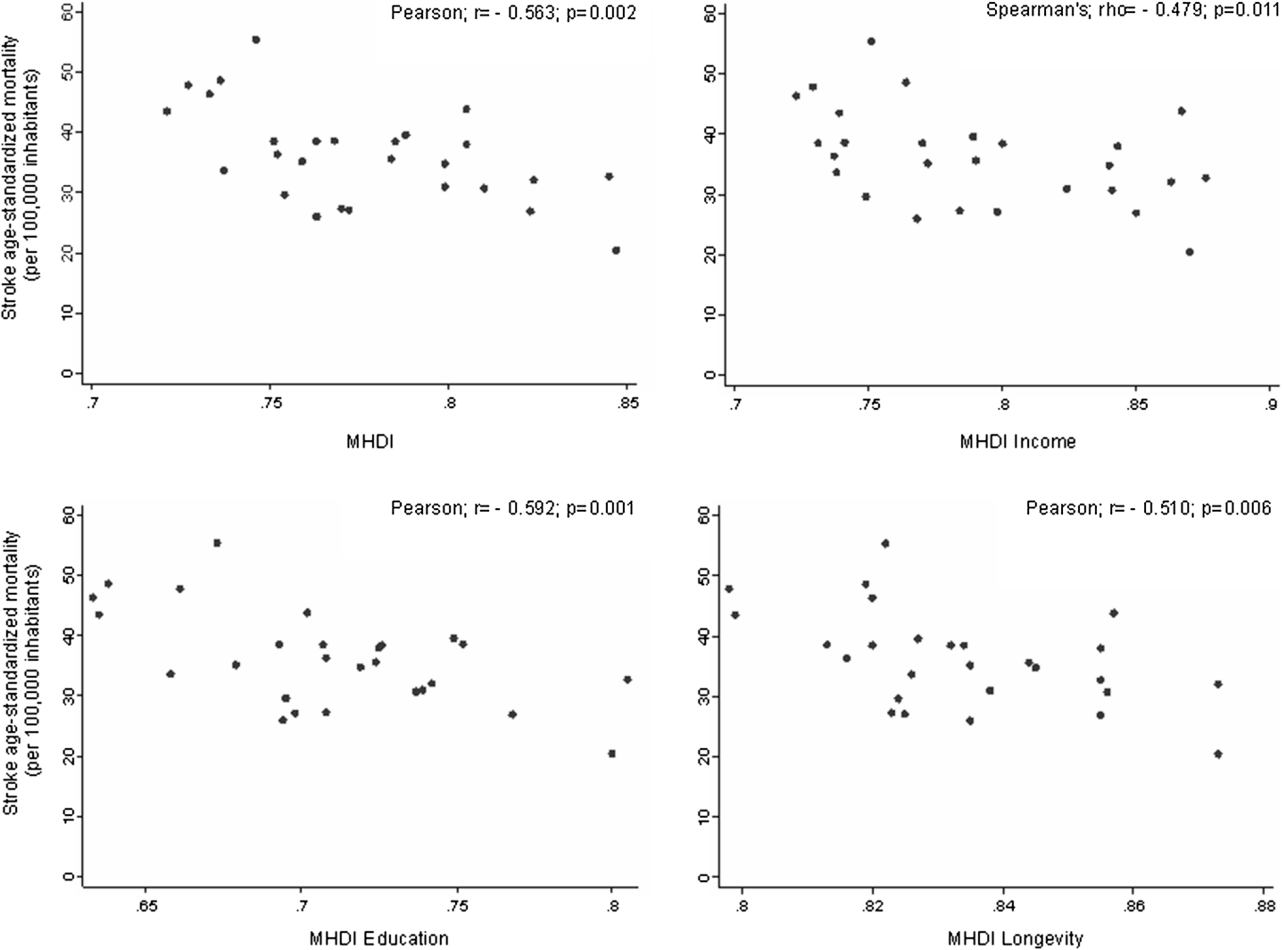
Relationship between human development indexes and stratification for income, education and longevity with stroke mortality in Brazilian capitals

The overall HDI presented a negative, moderate and significant correlation (Pearson r = − 0.593, p = 0.002) with age-standardized stroke mortality in Brazilian capitals. This correlation was also observed for the HDI dimensions, income (Spearman’s ρ = − 0.479, p = 0.011) education (Pearson r = − 0.592, p = 0.001) and longevity (Pearson r = − 0.510, p = 0.006) (Fig. 1).

### Discussion

In our analysis of the correlation between stroke mortality and municipal HDI, we observed a moderate and inverse correlation, which was also identified mainly between the education dimension of the MHDI and age-standardized stroke mortality.

Stroke is the second leading cause of death in industrialized countries and the leading cause of death in Brazil [17, 18]. Some risk factors for this disease, such as systemic arterial hypertension (SAH), obesity and hyperlipidemia, are related to unhealthy living standards [19]. Smoking is more often identified in populations of low socioeconomic status and nations with lower HDIs [20].

A negative correlation was identified between mortality due to stroke and income. This finding may be is because individuals with greater financial capacity receive more substantial and qualified health services, resulting in better treatment and adequate stroke prevention [21].

This Brazilian situation can be represented by Basic Health Units (*Unidade Básica de Saúde*—UBS), which are important in order for stroke risk factors, such as SAH and Diabetes Mellitus, to be controlled and prevented. Neto et al. demonstrated that regions with higher MHDIs had units with better services and high-quality infrastructure [22].

On the other hand, some studies have shown that countries with low and medium HDIs have different characteristics when compared with developed countries. In these nations, people of higher socioeconomic status have a higher risk of stroke death than do those of lower status [8].

Higher incomes may be associated with higher education, which may be associated with lower stroke mortality.

Thus, there are studies [11, 23] that have demonstrated, using longitudinal data, the presence of higher stroke mortality rates in individuals with low education levels. This pattern may be explained by the fact that individuals of higher socioeconomic status have better access to education, which may result in a better understanding of health, resulting in a lower stroke risk [21, 24].

At the same time, many studies have demonstrated an association between national educational development and various cancers incidence rates [25, 26], indicating that HDIs may play a role in non-communicable disease epidemiology.

Populations in regions with higher socioeconomic levels may have better access to education and qualified health services [27]. The availability of qualified health services facilitates and improves the possibility of early diagnosis of chronic diseases, such as cancer. These services may also reduce the lethality associated with these diseases, increasing population longevity [28].

Countries with higher HDIs may have a greater investment in health infrastructure and education and access to modern screening and treatment programs [27]. These measures may explain the longer life expectancies identified in these populations, even though age represents a risk factor for the development of cerebrovascular diseases [29].

Developing regions have been found to have higher rates of mortality from stroke. This finding may be explained by the gradual increase in life expectancy and increasingly westernized lifestyle [28, 30], which may be associated with habits such as smoking, alcohol consumption and physical inactivity [31].

The negative correlation identified between stroke and the socioeconomic indicator HDI was also observed in its dimensions: income, longevity and education.

## Limitations

This study has some limitations. The ecological data were susceptible to confounding, reverse causality and ecological fallacy and it is possible that associations at the individual level differ than at the group level. Stroke mortality data may suffer from some bias due the underestimation of mortality in data from the Mortality Information System (SIM/DATASUS). However, this system has been found to have good coverage [32, 33], which is estimated to be 7% by the proportion of poorly defined deaths [34].
